# Function of the Retinal Pigment Epithelium in Patients With Neurofibromatosis Type 1

**DOI:** 10.1167/iovs.63.4.6

**Published:** 2022-04-08

**Authors:** Romain Touzé, Marc M. Abitbol, Dominique Bremond-Gignac, Matthieu P. Robert

**Affiliations:** 1 Ophthalmology Department and Reference Center for Rare Ophthalmological Diseases (OPHTARA), AP-HP, University Hospital Necker–Enfants Malades, Paris, France; 2 Centre Borelli, ENS Paris-Saclay, Paris University, CNRS, INSERM, SSA, Paris, France; 3 INSERM, UMRS 1138, Team 17, From Physiopathology of Ocular Diseases to Clinical Development, Paris University, Paris, France

**Keywords:** genetic diseases, electrophysiology, retinal pigment epithelium

## Abstract

**Purpose:**

Retinal and choroidal abnormalities in neurofibromatosis type 1 (NF1) remain poorly studied. It has been reported, however, that the function of the retinal pigment epithelium (RPE) in NF1 was abnormal, with a supra-normal Arden ratio of the electro-oculogram (EOG). This study aims to evaluate the function of the RPE, using EOG, first in patients with NF1 compared to controls and second in patients with NF1 with choroidal abnormalities compared to patients with NF1 without choroidal abnormalities.

**Methods:**

This prospective case-control study included 20 patients with NF1 (10 patients with choroidal abnormalities and 10 patients without) and 10 healthy patients, matched for age. A complete ophthalmologic assessment with multimodal imaging, an EOG, and a full-field electroretinogram were performed for each included patient. The main outcome measured was the EOG light peak (LP)/dark trough (DT) ratio.

**Results:**

The LP/DT ratio was 3.02 ± 0.52 in patients with NF1 and 2.63 ± 0.31 in controls (*P* = 0.02). DT values were significantly lower in patients with NF1 than in controls (240 vs. 325 µV, *P* = 0.02), while light peak values were not significantly different (*P* = 0.26). No difference was found for peak latencies. No significant correlation between the surface and number of choroidal abnormalities and EOG parameters was demonstrated.

**Conclusions:**

This study confirms the dysfunction of the RPE in patients with NF1, involving a lower DT and a corresponding higher LP/DT ratio. We hypothesize that this pattern may be due to a dysregulation of the melanocytogenesis, inducing a disruption in Ca2^+^ ion flux and an abnormal polarization of the RPE.

Neurofibromatosis type 1 (NF1), also known as von Recklinghausen disease, is one of the most common autosomal dominant genetic diseases, with a mean estimated prevalence around 1:3000 in the world.^1^^,^^2^ Common manifestations of this disease involve skin, skeleton, central and peripheral nervous system, and eyes; they are notably represented by benign and malignant tumors.^1^ They result from dominant loss-of-function mutations of *NF1*, a tumor suppressor gene.^3^ The diagnosis relies on mostly clinical criteria, published in 1988 and revised in 2021.^4^^,^^5^

Two of these criteria—two or more iris Lisch nodules and optic pathway glioma (OPG)—are specifically detected by ophthalmologists, the latter having potential impact on the patients’ visual prognosis and a third of children with OPG showing visual deterioration.^6^ In addition, a new ophthalmologic criterion that can replace the presence of Lisch nodules has been added in the 2021 revision: the presence of two or more specific choroidal abnormalities. These abnormal images were first described by Yasunari et al.^7^ in 2000 on infrared pictures of the fundus. The prevalence of choroidal abnormalities in NF1 was estimated to be 80% in adults and 60% to 70% in the pediatric population, with a sensitivity of 70% to 80% and a high specificity reaching 95%.^8^^–^^12^ Choroidal abnormalities are easily seen on near-infrared (NIR) imaging and appear as bright patchy areas. On enhanced depth imaging–optic coherence tomography (EDI-OCT), these lesions seem to be localized in the deep layers of the choroid just below the choriocapillaris.^13^^,^^14^ However, the nature of these abnormalities still remains unknown. The reading of histologic reports published before the era of infrared imaging suggests that they could correspond to so-called ovoid bodies mainly composed of Schwann cells, ganglion cells, and/or melanocytes.^15^^,^^16^ As melanocytes increase the absorption of infrared light, this composition would be consistent with the hyperreflectance seen on infrared imaging.

Lubiński et al. first reported in 2001^17^ and then in 2004^18^ a dysfunction of the retinal pigment epithelium (RPE) in patients with NF1, with “supra-normal” electro-oculograms (EOGs). However, they stated that these changes were not correlated with any detectable changes in the fundus of the patients. Since then, choroidal abnormalities in NF1 have been reported and studied. We hypothesized that the pathologic composition of the RPE and choroid in NF1, reflected morphologically by these choroidal abnormalities, could be the cause of the electric activity changes of the RPE in NF1.

The primary goal of this study was to quantify the function of RPE, using EOGs, in patients with NF1 and in controls. The secondary goal was to study whether the presence of choroidal abnormalities would correlate with this dysfunction.

## Materials and Methods

### Study Design

This case-control study (NCT04153344) included prospectively 20 patients with NF1 and 10 healthy patients in our tertiary eye care center from April 2020 to September 2020. Files were collected in our institution using BaMaRa, a rare disease database.

### Population

The included patients had an NF1 diagnosis according to the National Institutes of Health criteria^4^ and were 7 years of age and older. The choice of including a young population of patients including children resulted from the study design with two subgroups of patients with NF1, considering that choroidal abnormalities appear over time and are largely present in NF1 adult populations.^19^ The group of patients with NF1 was subdivided into two subgroups, according to the presence of infrared hyperreflective choroidal abnormalities: 10 patients with choroidal abnormalities and 10 patients without choroidal abnormalities. Controls were 10 healthy patients, free from retinal or choroidal disorders and matched for age with patients from the NF1 group. All patients with other retinal diseases who had significant impairment of visual function or any disorder that restrained performing and analyzing an EOG or a full-field electroretinogram (ffERG) (significant oculomotor disorder, hyperactivity, poor compliance) were excluded.

### Clinical Examination

Each patient underwent a complete ophthalmic examination by two ophthalmologists (RT and MPR) in order to assess their refraction, including keratometry and axial length, their best-corrected visual acuity in logMAR, and the presence, nature, and/or number of any orbital abnormalities, Lisch nodules, and optic pathway gliomas.

### Electrophysiology

After pupil dilation by 1% tropicamide drops, EOG and ffERG were performed according to the International Society for Clinical Electrophysiology of Vision (ISCEV) recommendations.^20^^,^^21^ Pupil diameters were measured for each patient after dilation.

For EOG, a stimulus intensity equal to 100 candelas per square meter (cd⋅m^−2^) was used. First, the dark-trough (DT) amplitude (µV) and the DT latency (min), then the light-peak (LP) amplitude (µV) and the LP latency (min), were collected, so as to finally calculate the Arden index, also called the LP/DT ratio. This ratio, in patients and controls, consisted the primary outcome.

For each patient, EOG was always recorded first, followed by the recording of ffERG, in order to allow for interpretation of the EOG. Full-field ERGs were performed according to the ISCEV guidelines, after 20 minutes of dark adaptation. The dark-adapted (DA) responses to flash strengths of 0.01, 3.0, and 10.0 candela-seconds per meter squared (cd⋅s⋅m^–2^) were recorded: DA 0.01, DA 3.0, DA 10.0, and DA 3.0 oscillatory potentials. Then, a light adaptation of 10 minutes in a background with luminance of 30 cd⋅m^–2^ allowed us to record the light-adapted (LA) response to flash strengths of 3.0 cd⋅s⋅m^–2^ (LA 3.0). Finally, the LA flicker of 30 Hz was assessed with a 30-Hz flash stimulus at the luminance of 3.0 cd⋅s⋅m^–2^. Electrophysiologic exams were performed on the METROVISION *MonPackOne* (Metrovision, Perenchies, France) using Dawson, Trick, and Litzkow (DTL) electrodes. For each stimulation, amplitude and latency of a- and b-waves were collected. The entire study design is detailed in Supplemental Content 1.

### Retinal Imaging

In order to detect and quantify the choroidal abnormalities, each patient performed a 55-degree field near-infrared imaging, focused on the posterior pole, using a confocal scanning laser (HRAII or Spectralis HRA_OCT; Heidelberg Engineering, Heidelberg, Germany). For each patient and each eye, the number of patchy lesions was counted; each lesion was delimited, using manual segmentation provided by the area tool assistant included in the imaging viewer (Heyex Software; Heidelberg Engineering); and the total area of all choroidal abnormalities, observable in the 55-degree field, was measured.

Fundus pictures were made using the CR-2 PLUS AF, Digital Retinal Camera (Canon Medical Systems Europe, Zoetmeer, Netherlands). Subfoveal choroidal thickness was measured just behind the fovea with one horizontal high-definition line centered at the fovea using the EDI-OCT module of the Spectralis OCT (Spectralis HRA_OCT; Heidelberg Engineering).

### Data Analysis

The data analyses were performed with RStudio software (version 1.2.5033; RStudio, Inc., Boston, USA). Quantitative continuous variable results are presented as mean with standard deviation or median with range according to the distribution, and qualitative variables are presented by frequency (%). The χ^2^ or Fisher exact tests were performed for qualitative data. Quantitative variables were tested using a Mann–Whitney or a Student's *t*-test according to their distribution with a significance level of *P* < 0.05. The normal distribution of variables was tested using a Shapiro test. For data with values from both eyes, a mixed linear regression model was used in order to take into account data from both eyes and intraindividual correlations. Analyses were adjusted by age and gender. For all analyses, significant results were obtained with *P* < 0.05.

### Ethical Statement

This study (NCT04153344) was approved by the institutional review board CPP Île de France XI and conducted in accordance to the Declaration of Helsinki. Each participant provided written informed consent before inclusion in this study.

## Results

Twenty patients with NF1 and 10 age-matched controls were included. Demographic data and clinical characteristics of patients and controls are presented in Table 1.

**Table 1.** Patients’ Characteristics and Clinical and Ophthalmic Outcomes

	NF1 Group				
Characteristic	Total	With CAs	Without CAs	Control Group	*P* Value
Demographic data					
Patients, *n*	20	10	10	10	NA
Eyes, *n*	40	20	20	20	NA
Age, median [range], y	10 [8, 19]	10 [8, 19]	9 [8, 12]	11.9 [8, 16.4]	0.6
Male/female, *n*	7/20	5/5	2/8	6/4	1
Clinical and ophthalmic outcome					
Visual acuity, logMAR	0.0	0.0	0.0	0.0	1
Axial length, mean ± SD, mm	22.52 ± 1.16	22.50 ± 0.89	22.56 ± 1.55	22.90 ± 1.02	0.57
Lisch nodules, % (*n*)	65 (26/40)	70 (14/20)	60 (12/20)	NA	NA
Glioma, % (*n*)	35 (7/20)	30 (3/10)	40 (4/10)	NA	NA
Number of CAs/eye, median [range]	NA	10 [2, 21]	NA	NA	NA
Surface of CAs/eye, median [range], mm²	NA	10.42 [0.66, 57.64]	NA	NA	NA
Mean subfoveal choroidal thickness, mean ± SD, µm	340.7 ± 78	355.5 ± 57	324.6 ± 93	360.7 ± 64.3	0.46

BCVA, best-corrected visual acuity; CA, choroidal abnormality; NA, not applicable.

Visual acuity was normal (20/20) in each patient. Each group was comparable to the others for axial length, age distribution, and gender repartition. All patients exhibited a large pupil dilation with measured pupil diameters higher than 6.8 mm before electrophysiologic assessment.

**Figure 1.**
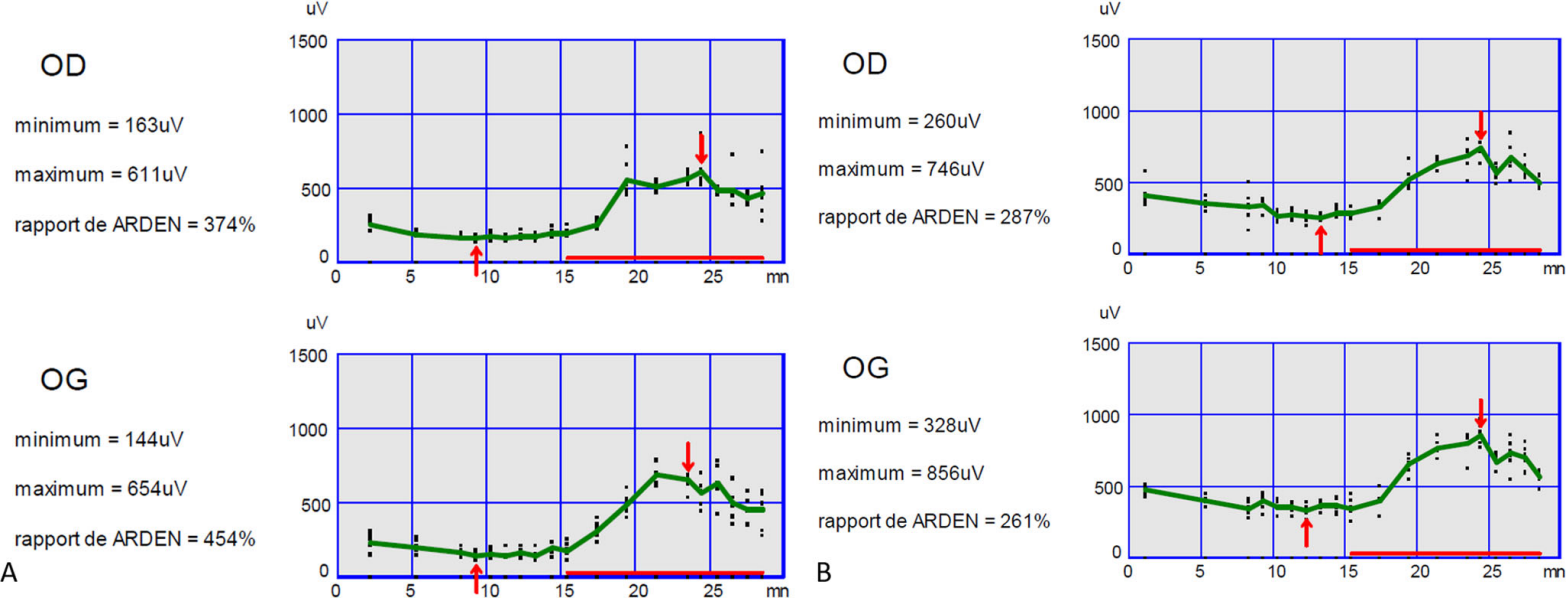
Typical supra-normal EOG results obtained in a child with NF1 (**A**) and a control (**B**). In the child with NF1, the dark trough occurred at 9 minutes with values of 163 µV for the right eye and 144 µV for the left eye, and the light peak occurred at 23 to 24 minutes with values of 611 µV for the right eye and 654 µV for the left eye with an LP/DT ratio of 3.74 and 4.54, respectively. In the healthy child, the dark trough occurred at 12 minutes with values of 260 µV for the right eye and 328 µV for the left eye, and the light peak occurred at 23 to 24 minutes with values of 746 µV for the right eye and 856 µV for the left eye with an LP/DT ratio of 2.87 and 2.61, respectively. OD, oeil droit meaning right eye; OG, oeil gauche meaning left eye.

**Figure 2.**
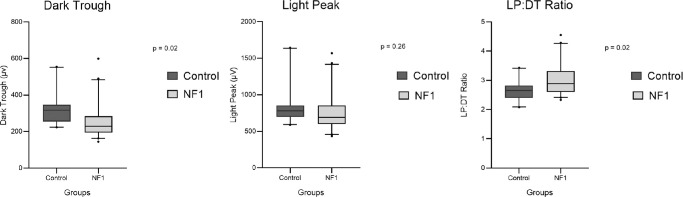
Boxplot of electro-oculogram values obtained between patients with NF1 and controls. Each value, including LP, DT, and LP/DT ratio, is represented. The first quartile is the *bottom* of the *box* and the third quartile the *top* of the *box*. The *center line* represents the median value; *whiskers* include the 5th to 95th percentiles of data; outliers are represented as *black dots*. *P* values are indicated above.

The ERG responses were normal in each eye of each included patient, allowing for a proper interpretation of EOG responses. The mean LP/DT ratio was 3.02 ± 0.52 in patients and 2.63 ± 0.32 in controls (*P* = 0.02). Supra-normal EOG with a LP/DT ratio above the upper limit of normal (LP/DT mean + 2 SD – >3.08) was found in 50% (10/20) of all patients with NF1 (see example in Fig. 1). No difference for DT and LP latencies was found. DT amplitude values were significantly lower in patients with NF1 than in controls (*P* = 0.02), but no statistical difference was found for LP amplitudes (*P* = 0.26) (Table 2 and Fig. 2).

**Table 2.** Electro-Oculogram Results

			*P* (NF1	*P* (NF1 with	*P* (NF1 with
Characteristic	Number	Mean ± SD	vs. Control)	CAs vs. without CAs)	CAs vs. Control)
Dark trough, µV					
** **NF1	20	240 ± 76	0.02^*^	0.9	<0.01
** **NF1 with CAs	10	239 ± 58			
** **NF1 without CAs	10	241 ± 94			
** **Control	10	325 ± 87			
Light peak, µV					
** **NF1	20	747 ± 236	0.26	0.8	0.16
** **NF1 with CAs	10	718 ± 178			
** **NF1 without CAs	10	776 ± 288			
** **Control	10	850 ± 260			
LP/DT ratio					
** **NF1	20	3.02 ± 0.52	0.02^*^	0.72	0.01
** **NF1 with CAs	10	3.03 ± 0.54			
** **NF1 without CAs	10	3.01 ± 0.58			
** **Control	10	2.63 ± 0.31			

* Statistically significant (with mixed linear regression model).

In the subgroup of patients with NF1 with choroidal abnormalities, DT (239 ± 58 µV) and LP (718 ± 178 µv) mean values were lower than in the subgroup of patients with NF1 without choroidal abnormalities, but this difference was not significant (*P* = 0.9 and 0.8, respectively). No correlation was found between surface and number of choroidal abnormalities and values of EOG (using mixed-model regression and Pearson linear regression).

**Figure 3.**
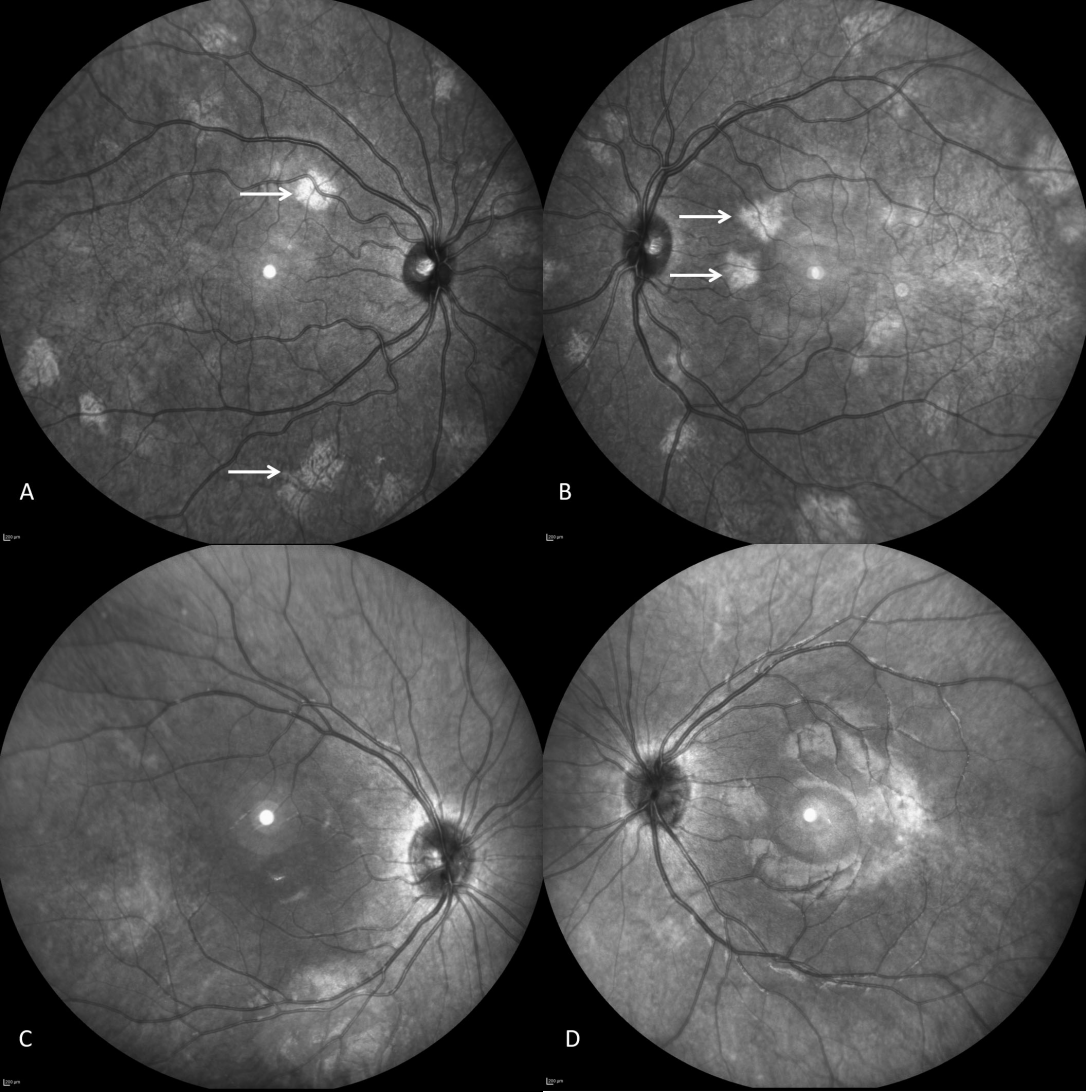
Near-infrared imaging fundus in a 12-year-old boy with NF1: (**A**) right eye and (**B**) left eye. Typical patchy bright lesions were observed in both eyes, representing typical choroidal abnormalities (*white arrow*). Area of these abnormalities was 12.18 mm² on the right eye and 11.36 mm² on the left eye. Normal near-infrared imaging fundus in a healthy 11-year-old girl is presented for comparison: (**C**) right eye and (**D**) left eye.

**Figure 4.**
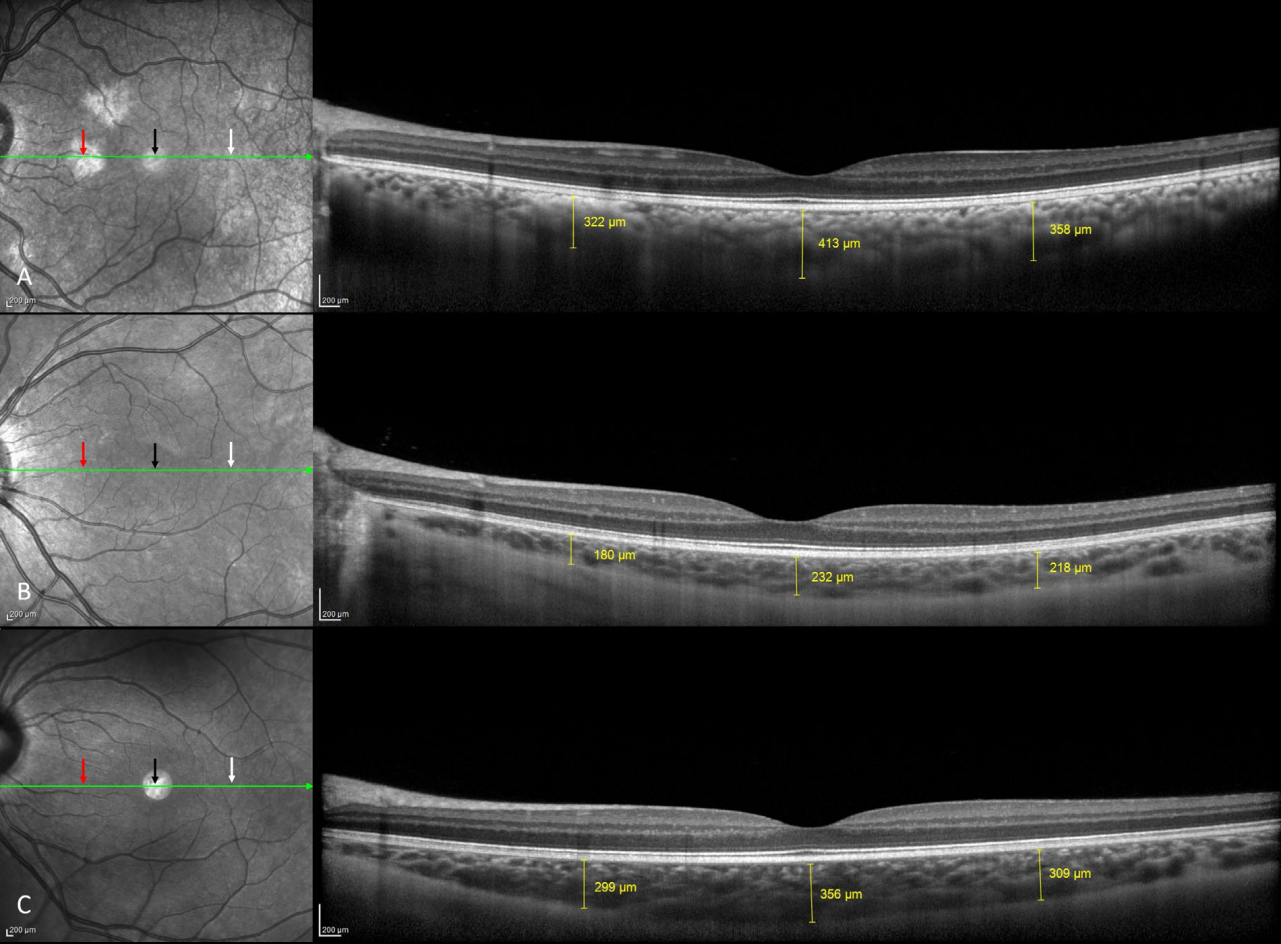
Comparative spectral-domain optical coherence tomography scans with EDI in children with NF1 and choroidal abnormalities (**A**), without choroidal abnormalities (**B**), and without NF1 (**C**). The subfoveal choroidal thickness was assessed in each patient in a single point located behind the fovea (*black arrow*). These scans compared choroidal thickness of a choroidal abnormality (*red arrow*, **A**) and correspondent area in a patient with NF1 but without a choroidal abnormality (*red arrow*, **B**) and control (*red arrow*, **C**). The opposite choroidal thickness, 2000 µm from the fovea temporally, was measured (*white arrow*). No statistical difference of choroidal thickness was observed between groups.

In the subgroup with choroidal abnormalities, the median number of abnormalities was 10 (2–21) for a median area of 10.6 (0.66–57.64) mm^2^ per eye (see example in Fig. 3^4^). The subfoveal choroidal thickness, measured with the EDI-OCT line, was 355.5 ± 57 µm, compared to 360.7 ± 64.3 µm in control patients (*P* = 0.46) (see example in Fig. 4). No significant difference was found compared to the group without choroidal abnormalities or to the control group (Table 1).

## Discussion

Only one group has published data related to the function of RPE in patients with NF1.^17^^,^^18^ They found a significant supra-normal EOG with a higher LP/DT ratio in patients with NF1 versus controls: 3.42 ± 0.62 vs. 2.45 ± 0.37 (*P* < 0.01). These results had not been replicated so far. In the present study, the LP/DT ratio was 3.02 ± 0.52 in patients with NF1 versus 2.63 ± 0.31 in age-matched controls (*P* = 0.02). As in the present study, Lubiński et al.^17^^,^^18^ had found a significantly lower DT amplitude in patients with NF1 compared to healthy controls: 305.0 ± 81.2 μV versus 408.1 ± 112.9 μV, respectively (*P* < 0.01), but no statistical difference for the LP amplitude between patients with NF1 and controls. The increase of the LP/DT ratio mainly resulted from the decrease of the DT amplitude. Our study confirms these previously published data, but the reasons explaining these results remain uncertain.

The RPE is a unique unicellular layer, essential to the function of the choroid/neuroretina complex. In interacting with the outer segments of the photoreceptors via the apical membrane and with Bruch's membrane via the basolateral membrane, the RPE is involved in light absorption; in water, ion, and nutrient transport; in the visual cycle; in the phagocytosis of shed outer segments of photoreceptors; and in the production of growth factors; finally, the RPE is part of the blood/retina barrier.^22^

The RPE apical membrane is positively polarized, while the basolateral membrane is negatively polarized, resulting in a differential potential called the transepithelial potential. The EOG allows one to measure this potential spontaneously generated during dark adaptation (the DT value, also called the resting potential); then the voltage difference increases with light stimulation (the LP value); this results from a depolarization of the RPE basolateral membrane, involving ionic gradients.^23^ A high intracellular concentration of Cl^–^ provides the positive transepithelial potential. A dysregulation of the intracellular Ca2^+^ in the RPE would induce alteration of the differential potential via Ca2^+^-dependent Cl^–^ channels regulating Cl^–^ intracellular concentration.^22^ Such an alteration would explain the EOG values found in this study: a low DT with a preserved, normal (i.e., relatively high) LP. Yet, it is acknowledged that Ca2^+^ is contained in melanin pigment. Besides their light protection role, melanin pigments bind many chemical elements, such as Ca2^+^.^24^ Melanosomes are therefore important in organellar Ca2^+^ storage and are key in the RPE intracellular concentration of Ca2^+^.^25^^–^^27^

The skin and choroid melanocytes of patients with NF1 contain more melanocytes, more melanin pigment, and a high density of enlarged melanin granules called macromelanosomes.^28^^–^^30^ These are likely not true melanosomes (which are secretory lysosomes) but actual autolysosomes, in which formation results from the dysregulation in melanocytogenesis. It has recently been shown that the *NF1* gene could interact with dynein heavy chain 1 (DHC1) and induce mislocalization of melanosomes to the distal tips of melanocytes in the choroid.^28^ It is likely that the RPE in NF1 is involved in the very same way as the underlying choroid.

Other pathologic conditions characterized by abnormal EOG values may cast light on what is observed in patients with NF1. In Best disease, EOG responses exhibit an absence of genesis of the light peak. Recent studies have suggested that the RPE dysfunction in Best disease results from a disruption in Ca2^+^-dependent Cl^–^ channels through intracellular Ca2^+^ signaling.^31^ Interestingly, two studies also recorded EOG in patients with ocular albinism (OA) and oculo-cutaneous albinism (OCA) and found a normal or supra-normal LP/DT ratio with significantly low DT amplitudes,^32^^,^^33^ strikingly similar to the pattern found in patients with NF1, whereas albinism could appear as the clinical opposite of NF1. The authors proposed that these results could be explained by the abnormal melanosomal biogenesis and light-induced retinal damage.^33^^,^^34^ A dysregulation of melanocytogenesis is observed in OA,^35^^,^^36^ where the ocular hypopigmentation results from a disruption in melanosome organellogenesis rather than from an abnormal melanin synthesis, as is the case in OCA.^37^ Remarkably, macromelanosomes are also observed in patients with OA.^36^^,^^38^

We hypothesize that the disruption in melanocytogenesis observed in patients with NF1 results in an RPE dysfunction characterized by low DT amplitudes and supra-normal EOG. Disruption in melanocytogenesis and abnormal macromelanosomes are present in both patients with NF1 and patients with OA, who both exhibit a similar EOG pattern, which likely results from a comparable disruption in Ca2^+^ ion flux. As little is known about RPE structure and function in NF1, it is not possible so far to rule out alternative mechanisms leading to an abnormal polarization of the RPE in NF1.

One of the main limitations of this study relates to the variability of values obtained during electrophysiologic assessment. In order to limit this variability, ISCEV guidelines were strictly followed.^20^^,^^21^ We excluded all patients with clinical parameters that could have artifacted the obtained responses. All examinations were performed in the same room, by the same practitioner (RT), using the same devices and following the same procedure. It is known that the LP/DT ratio tends to decrease with age. Thavikulwat et al.^39^ found a decrease of 0.13 by decades. In this study, both groups were matched by age without a difference in the distribution. Moreover, in light of the patients’ ages, comprised within only one decade, this bias can reasonably be excluded.

No difference was observed in choroidal thickness between both groups, with and without CA. One reason could be the method used to assess choroidal thickness, measured using EDI-OCT, in a single point behind the fovea. In patients with CA, however, the relation between choroidal thickness behind the fovea and across the retina might differ from normal. A large future study comparing mean choroidal thickness across the retina in patients with CA and controls would be interesting.

No significant correlation was found between the LP/DT ratio and the surface of choroidal abnormalities. Obviously, the mean surface of pictured choroidal abnormalities—10.42 mm² per eye in the NF1 group with CA—is negligible compared to the total surface of the retina—about 1300 mm².^40^ Electro-oculography investigates the function of the whole RPE. Choroidal abnormality areas, however, were measured on 55° field imaging and not ultra-wide field imaging. Future studies should ideally use the latter in order not to overlook choroidal abnormalities beyond the central 55°.

In the subgroup with choroidal abnormalities, the values of DT and LP were lower than in the subgroup without choroidal abnormalities, although this did not reach significance; future studies could study a larger retinal area in a greater number of patients in order to increase the power of the study. We do not hypothesize any direct effect of choroidal abnormalities on EOG, but rather that in NF1, choroidal abnormalities would be the anatomic marker of an underlying functional abnormality, which itself would be responsible for the supra-normal Arden ratio.

Another reason for which a correlation between the choroidal abnormalities and the RPE function may have been overlooked results from the study design itself: as choroidal abnormalities appear over time, included patients were young, and as EOG responses evolve with age, it was crucial to age-match the groups of participants. However, it may be that the RPE dysfunction also increases with age and that such a correlation requires older populations, and/or a longitudinal study, to be performed.

Additionally, the role of the protein HTRA1, recently involved in the pathogenesis of AMDs and other diseases of the RPE,^41^ and the interactions between choroidal vessels deserve certainly further investigations.

This study confirms that patients with NF1 exhibit a supra-normal Arden ratio on EOG, which results from low DT amplitudes. We hypothesize that these responses are explained by a dysregulation of melanocytogenesis, inducing abnormal polarization on both sides of RPE (apical and basal membranes).

## Supplementary Material

Supplement 1

## References

[1] Gutmann DH, Ferner RE, Listernick RH, Korf BR, Wolters PL, Johnson KJ. Neurofibromatosis type 1. *Nat Rev Dis Primer*. 2017; 3(1): 17004. 10.1038/nrdp.2017.4 28230061

[2] Uusitalo E, Leppävirta J, Koffert A, et al. Incidence and mortality of neurofibromatosis: a total population study in Finland. *J Invest Dermatol*. 2015; 135(3): 904–906. 25354145 10.1038/jid.2014.465

[3] Wallace MR, Marchuk DA, Andersen LB, et al. Type 1 neurofibromatosis gene: identification of a large transcript disrupted in three NF1 patients. *Science*. 1990; 249(4965): 181–186. 2134734 10.1126/science.2134734

[4] Neurofibromatosis. Conference statement. National Institutes of Health consensus development. *Arch Neurol*. 1988; 45: 575–578. 3128965

[5] Legius E, Messiaen L, Wolkenstein P, et al. Revised diagnostic criteria for neurofibromatosis type 1 and Legius syndrome: an international consensus recommendation. *Genet Med*. 2021; 23(8): 1506–1513. 34012067 10.1038/s41436-021-01170-5 PMC8354850

[6] Listernick R, Charrow J, Greenwald M, Mets M. Natural history of optic pathway tumors in children with neurofibromatosis type 1: a longitudinal study. *J Pediatr*. 1994; 125(1): 63–66. 8021787 10.1016/s0022-3476(94)70122-9

[7] Yasunari T, Shiraki K, Hattori H, Miki T. Frequency of choroidal abnormalities in neurofibromatosis type 1. *Lancet*. 2000; 356(9234): 988–992. 11041400 10.1016/S0140-6736(00)02716-1

[8] Viola F, Villani E, Natacci F, et al. Choroidal abnormalities detected by near-infrared reflectance imaging as a new diagnostic criterion for neurofibromatosis 1. *Ophthalmology*. 2012; 119(2): 369–375. 21963267 10.1016/j.ophtha.2011.07.046

[9] Nakakura S, Shiraki K, Yasunari T, Hayashi Y, Ataka S, Kohno T. Quantification and anatomic distribution of choroidal abnormalities in patients with type I neurofibromatosis. *Graefes Arch Clin Exp Ophthalmol*. 2005; 243(10): 980–984. 15891894 10.1007/s00417-005-1184-z

[10] Parrozzani R, Clementi M, Frizziero L, et al. In vivo detection of choroidal abnormalities related to NF1: feasibility and comparison with standard NIH diagnostic criteria in pediatric patients. *Invest Ophthalmol Vis Sci*. 2015; 56(10): 6036–6042. 26393470 10.1167/iovs.14-16053

[11] Vagge A, Camicione P, Capris C, et al. Choroidal abnormalities in neurofibromatosis type 1 detected by near-infrared reflectance imaging in paediatric population. *Acta Ophthalmol (Copenh)*. 2015; 93(8): e667–e671. 10.1111/aos.12750 25990002

[12] Moramarco A, Giustini S, Nofroni I, et al. Near-infrared imaging: an in vivo, non-invasive diagnostic tool in neurofibromatosis type 1. *Graefes Arch Clin Exp Ophthalmol*. 2018; 256(2): 307–311. 29290016 10.1007/s00417-017-3870-z

[13] Rao RC, Choudhry N. Enhanced depth imaging spectral-domain optical coherence tomography findings in choroidal neurofibromatosis. *Ophthalmic Surg Lasers Imaging Retina*. 2014; 45(5): 466–468. 25153660 10.3928/23258160-20140818-01 PMC4398345

[14] Kumar V, Singh S. Multimodal imaging of choroidal nodules in neurofibromatosis type-1. *Indian J Ophthalmol*. 2018; 66(4): 586–588. 29582830 10.4103/ijo.IJO_1095_17 PMC5892072

[15] Wolter JR. Nerve fibrils in ovoid bodies: with neurofibromatosis of the choroid. *Arch Ophthalmol*. 1965; 73(5): 696–699. 14281990 10.1001/archopht.1965.00970030698019

[16] Ueda-Consolvo T, Miyakoshi A, Ozaki H, Houki S, Hayashi A. Near-infrared fundus autofluorescence-visualized melanin in the choroidal abnormalities of neurofibromatosis type 1. *Clin Ophthalmol*. 2012; 6: 1191–1194. 22888215 10.2147/OPTH.S35321 PMC3413334

[17] Lubiński W, Zajaczek S, Sych Z, Penkala K, Palacz O, Lubiński J. Electro-oculogram in patients with neurofibromatosis type 1. *Doc Ophthalmol Adv Ophthalmol*. 2001; 103(2): 91–103. 10.1023/a:1012271206258 11720259

[18] Lubiński W, Zajączek S, Sych Z, Penkala K, Palacz O, Lubiński J. Supernormal electro-oculograms in patients with neurofibromatosis type 1. *Hered Cancer Clin Pract*. 2004; 2(4): 193–196. 20233463 10.1186/1897-4287-2-4-193 PMC2840006

[19] Touzé R, Manassero A, Bremond-Gignac D, Robert MP. Long-term follow-up of choroidal abnormalities in children with neurofibromatosis type 1. *Clin Experiment Ophthalmol*. 2021; 49(5): 516–519. 33893699 10.1111/ceo.13936

[20] Constable PA, Bach M, Frishman LJ, Jeffrey BG, Robson AG. ISCEV standard for clinical electro-oculography (2017 update). *Doc Ophthalmol Adv Ophthalmol*. 2017; 134(1): 1–9. 10.1007/s10633-017-9573-2 PMC5309273 28110380

[21] McCulloch DL, Marmor MF, Brigell MG, et al. ISCEV standard for full-field clinical electroretinography (2015 update). *Doc Ophthalmol*. 2015; 130(1): 1–12. 10.1007/s10633-014-9473-7 25502644

[22] Strauss O. The retinal pigment epithelium in visual function. *Physiol Rev*. 2005; 85(3): 845–881. 15987797 10.1152/physrev.00021.2004

[23] Sparrow JR, Hicks D, Hamel CP. The retinal pigment epithelium in health and disease. *Curr Mol Med*. 2010; 10(9): 802–823. 21091424 10.2174/156652410793937813 PMC4120883

[24] Schraermeyer U, Heimann K. Current understanding on the role of retinal pigment epithelium and its pigmentation. *Pigment Cell Res*. 1999; 12(4): 219–236. 10454290 10.1111/j.1600-0749.1999.tb00755.x

[25] Wiriyasermkul P, Moriyama S, Nagamori S. Membrane transport proteins in melanosomes: regulation of ions for pigmentation. *Biochim Biophys Acta Biomembr*. 2020; 1862(12): 183318. 32333855 10.1016/j.bbamem.2020.183318 PMC7175901

[26] Panessa BJ, Zadunaisky JA. Pigment granules: a calcium reservoir in the vertebrate eye. *Exp Eye Res*. 1981; 32(5): 593–604. 6972313 10.1016/s0014-4835(81)80008-5

[27] Salceda R, Sánchez-Chávez G. Calcium uptake, release and ryanodine binding in melanosomes from retinal pigment epithelium. *Cell Calcium*. 2000; 27(4): 223–229. 10858668 10.1054/ceca.2000.0111

[28] Arun V, Worrell L, Wiley JC, Kaplan DR, Guha A. Neurofibromin interacts with the cytoplasmic dynein heavy chain 1 in melanosomes of human melanocytes. *FEBS Lett*. 2013; 587(10): 1466–1473. 23583712 10.1016/j.febslet.2013.03.035

[29] Martuza RL, Philippe I, Fitzpatrick TB, Zwaan J, Seki Y, Lederman J. Melanin macroglobules as a cellular marker of neurofibromatosis: a quantitative study. *J Invest Dermatol*. 1985; 85(4): 347–350. 3930616 10.1111/1523-1747.ep12276952

[30] Wolter JR, Gonzales-Sirit R, Mankin WJ. Neurofibromatosis of the choroid. *Am J Ophthalmol*. 1962; 54(2): 217–225. 14040313

[31] Cordes M, Bucichowski P, Alfaar AS, et al. Inhibition of Ca2+ channel surface expression by mutant bestrophin-1 in RPE cells. *FASEB J*. 2020; 34(3): 4055–4071. 31930599 10.1096/fj.201901202RR

[32] Gahlot DK, Hansen E. Electro-oculography in albinos. *Acta Ophthalmol (Copenh)*. 1974; 52(2): 220–224. 4406781 10.1111/j.1755-3768.1974.tb00370.x

[33] Reeser F, Weinstein GW, Feiock KB, Oser RS. Electro-oculography as a test of retinal function. *Am J Ophthalmol*. 1970; 70(4): 505–514. 5505468 10.1016/0002-9394(70)90883-4

[34] Nusinowitz S, Sarraf D. Retinal function in X-linked ocular albinism (OA1). *Curr Eye Res*. 2008; 33(9): 789–803. 18798082 10.1080/02713680802376353

[35] McKay BS. Pigmentation and vision: is GPR143 in control? *J Neurosci Res*. 2019; 97(1): 77–87. 29761529 10.1002/jnr.24246 PMC6235735

[36] Cortese K, Giordano F, Surace EM, et al. The ocular albinism type 1 (OA1) gene controls melanosome maturation and size. *Invest Opthalmol Vis Sci*. 2005; 46(12): 4358. 10.1167/iovs.05-0834 16303920

[37] Grønskov K, Ek J, Brondum-Nielsen K. Oculocutaneous albinism. *Orphanet J Rare Dis*. 2007; 2(1): 43. 17980020 10.1186/1750-1172-2-43 PMC2211462

[38] Garner A, Jay BS. Macromelanosomes in X-linked ocular albinism. *Histopathology*. 1980; 4(3): 243–254. 7390409 10.1111/j.1365-2559.1980.tb02919.x

[39] Thavikulwat AT, Lopez P, Caruso RC, Jeffrey BG. The effects of gender and age on the range of the normal human electro-oculogram. *Doc Ophthalmol Adv Ophthalmol*. 2015; 131(3): 177–188. 10.1007/s10633-015-9514-x PMC4749427 26474906

[40] Nagra M, Gilmartin B, Thai NJ, Logan NS. Determination of retinal surface area. *J Anat*. 2017; 231(3): 319–324. 28620965 10.1111/joa.12641 PMC5554828

[41] Williams BL, Seager NA, Gardiner JD, et al. Chromosome 10q26–driven age-related macular degeneration is associated with reduced levels of HTRA1 in human retinal pigment epithelium. *Proc Natl Acad Sci USA*. 2021; 118(30): e2103617118. 34301870 10.1073/pnas.2103617118 PMC8325339

