# Supplementary material for: Function of the Retinal Pigment Epithelium in Patients With Neurofibromatosis Type 1
Source: Invest Ophthalmol Vis Sci. 2022 Apr 8;63(4):6. doi: 10.1167/iovs.63.4.6 (PMC8994170; doi:10.1167/iovs.63.4.6)
Supplement: Supplement 1 [file iovs-63-4-6_s001.pdf]

Supplemental content 1

Protocol of electro-oculogram

All included patients were examined in the same condition, in the same electrophysiology room and with the same material. Each patient was prepared during 30 minutes of adaptation in a stable indoor lighting ambiance. During this period, all important source of light as others examinations were avoided. The pupils were dilated with tropicamide 1% drops to obtain a full dilation. Pupils' diameters were collected. Skin electrodes (Comepa industries, Bagnolet, France) were placed, after skin preparation, close to the lateral and medial canthi of each eye and connected on separate channels of the amplifier. One forehead skin electrode was attached and connected to the ground. The simulator used was the METROVISION *MonPackOne* (Metrovision, Lille, France). Saccades were generated using fixation red lights on the screen appearing alternatively on the right and on the left once per second, for 10 seconds every minute. Each EOG potentials were recorded during these 10 seconds periods. The first phase recorded was the dark phase, with total darkness maintained during 15 minutes. Saccades recorded during this phase were smoothed to obtain the dark trough (DT) amplitude in µV. Then a light phase was initiated with a ganzfeld background light of 100 $cd.m^{-2}$ turned on during 15 minutes to obtain the light peak (LP) amplitude in µV as smoothed EOG potentials during this phase. Latencies of DT and LP were also recorded in minutes. The LP:DT ratio was obtained by dividing the LP by the DT amplitudes.

Protocol of full field electroretinogram

All included patients were examined in the same condition, in the same electrophysiology room and with the same material. The pupils were dilated with tropicamide 1% drops to obtain a full dilation. Pupils' diameters were collected. Eye contact electrodes as silver thread ERG electrodes (Medical Physics & Clinical Engineering, Liverpool, UK), connected to the positive input, have been used after topical anaesthesia with oxybuprocaine 0.4% drops. Two reference electrodes (Comepa industries, Bagnolet, France), connected to the negative input, were placed in each lateral canthus. The common electrode (Comepa industries, Bagnolet, France), connected to the ground, were placed on forehead. The simulator used was the METROVISION *MonPackOne* (Metrovision, Lille, France). After a dark adaptation of 20 minutes, the following sequences of stimulation were recorded according to the ISCEV guidelines:

- Dark-adapted 0.01 cd.s.m^{-2} (DA 0.01) ERG
- Dark-adapted 3.0 cd.s.m^{-2} (DA 3.0) ERG
- Dark-adapted 10.0 cd.s.m^{-2} (DA 10.0) ERG
- Dark-adapted oscillatory potentials (DA 3.0 OPs)

After a period of 10 minutes light adaptation with a ganzfeld background of 30 cd.s.m^{-2}, the light-adapted sequences were recorded:

- Light-adapted 3.0 cd.s.m^{-2} (LA 3.0) ERG
- Light-adapted 30 Hz flicker ERG, consisting in train of flash during less than 5 ms, presented at a rate of 30 stimuli by seconds (30Hz).

Amplitudes and peak times of the a- and b-waves were measured for each response.
